# Translation and validation of the simplified Chinese new Knee Society Scoring System

**DOI:** 10.1186/s12891-015-0854-1

**Published:** 2015-12-21

**Authors:** Denghui Liu, Xiaokang He, Wei Zheng, Yu Zhang, Dahe Li, Wei Wang, J. Li, Weidong Xu

**Affiliations:** Department of Orthopedics, Changhai Hospital, Second Military Medical University, 168 Changhai Road, Shanghai, 200433 China

**Keywords:** Simplified Chinese version, New society knee scoring system, Questionnaire, Validity, Reliability

## Abstract

**Background:**

The NKSS has recently been translated into Dutch version. The reliability and validity were also assessed. However, there is no Simplified Chinese version of New Society Knee Scoring System (SC-NKSS) for Chinese population.

**Methods:**

The SC-NKSS was translated from the original English version following international guidelines. All patients undergoing total knee arthroplasty (TKA) between September 2012 and September 2013 were invited to participate in this study. Finally, a total of 105 did so. Patients (preoperative and postoperative) completed the Chinese version of NKSS, Oxford Knee Score (OKS), the Medical Outcomes General Health Survey (SF-36) and Visual analog scale (VAS). Psychometric testing of reliability, construct validity, content validity were conducted.

**Results:**

All the 105 participants completed the questionnaires and no floor or ceiling effects were checked. Internal consistency was excellent with Cronbach’s alpha coefficient ranging from 0.71 to 0.85. Test-retest reliability was satisfactory with an intraclass correlation coefficient of 0.92 (95%confidence interval, 0.86–0.95). Construct validity was demonstrated to correlate well with the Chinese version of OKS (*r* =−0.78; *p* < 0.01), VAS (*r* =−0.70; *p* < 0.01), Physical Function (PF) (*r* = 0.74; *p* < 0.01), Body Pain (BP) (*r* = 0.63; *p* < 0.01) and General Health (GH) (*r* = 0.51; *p* < 0.01) of SF-36 domains.

**Conclusion:**

The SC-NKSS was well accepted and demonstrated acceptable psychometric properties in mainland China.

**Electronic supplementary material:**

The online version of this article (doi:10.1186/s12891-015-0854-1) contains supplementary material, which is available to authorized users.

## Background

Osteoarthritis (OA) is the most common joint disease and a major public health problem throughout the world [1, 2]. Since the publication of the first studies of total knee arthroplasty (TKA) in the 1960s, TKA has developed into a reliable surgical procedure to reduce pain, restore mobility and improve the quality of life for patients with osteoarthritis of the knee [3, 4]. Meanwhile, many questionnaires for evaluating knee joint function have appeared [5]. In 1989, The Knee Society Clinical Rating System was developed, and it became the most prevalent method to track and assess outcomes after TKA throughout the world [6, 7]. However, with the improvement of equipment and surgical techniques, the age range of patients receiving TKA has expanded [3]. Besides, the OA knee patients have high expectation and want excellent knee function by TKA. As a result, the reliability, validity and responsiveness of the Knee Society Clinical Rating System have been challenged [2, 6].

Recently, the NKSS has been designed to be completed by both physicians and patients [6]. The objective section of the NKSS, which includes the technical outcome of the procedure on the basis of pain, range of motion (ROM), alignment and stability, is completed by the physician, and the subjective parameters of the NKSS, which include patients’ knee function, satisfaction and fulfilment of expectations, is completed by the patient [1, 6]. The internal consistency, construct and convergent validity and reliability of the NKSS have been confirmed by Noble PC. etc [8] using statistical analysis in the United States and Canada. The NKSS has been cross-culturally adapted into Dutch [9] and has demonstrated satisfactory psychometric properties when applied to patients with TKA.

It is well known that TKA is the most effective intervention to improve the quality of life for patients with the terminal stage of osteoarthritis [5]. China has the largest population in the world, and the prevalence ratio of radiographic knee OA is 42.8 % in women, 21.5 % in men. Symptomatic OA knee occurred in 15.0 % of women and 5.6 % of men in Beijing. In mainland China, rural areas have a higher incidence f symptomatic OA knee than urban regions [10, 11]. Many assessment questionnaires have been translated into simplified Chinese versions, including the Oxford Knee Score [12] and Visual Analogue Score [13, 14]. However, there is no so-called “gold standard” that optimally evaluates the outcome of TKA [15, 16]. Therefore, development of the SC-NKSS may provide more choice for doctors or physicians to evaluate clinical outcomes of patients with OA knee. The aim of this study was to translate and cross-culturally adapt the NKSS into a simplified Chinese version and to evaluate its psychometric properties, and we specially tested the reliability, validity of the SC-NKSS.

## Methods

### Translation and cross-cultural adaptation

The translation and cross-cultural adaptation of the original NKSS were performed according to international guidelines [17, 18] (Additional file 1). First, the original NKSS was translated into first Chinese version by two bilingual translators who were native Chinese, one (author of the article, Denghui Liu) has medical background, the other without knowing the study purpose and does not have medical background. Second, synthesize the translation, the discrepancies of the first Chinese version were resolved by reaching a consensus of two translators. Third, backward translation of the first Chinese version was performed by two independent native English speakers with Chinese as their second language (David Sorenson, William Marks), they have medical background and were blind to the study purpose, and reached a consensus on backward translation. Fourth, we established an expert committee composed of four translators, two orthopaedic surgeons, one physician of rehabilitation, and one physical therapist. The backward translation was compared with the first Chinese version and original version by expert committee. The committee reached agreement on the semantic, idiomatic, experiential and conceptual equivalence between the original and the target versions. Finally, the agree-upon version was pre-tested on 15 patients who were ready to undergo TKA and 15 patients who had already undergone TKA. A few patients had difficulties understanding the simplified Chinese items because of the semantic, idiomatic, experiential and conceptual differences. To help patients understand the questionnaire items, the investigators met them in the outpatient or inpatient departments and made note of the difficult items, which would be discussed by the expert committee and replaced with more appropriate simplified Chinese characters. Finally, these pre-tested patients can complete the questionnaires [19]. The expert committee reached consensus on the final version which was then subjected to further psychometric testing.

### Patients

All patients undergoing total knee arthroplasty (TKA) were invited to participate in this study. A total of 105 patients with a diagnosis of knee osteoarthritis were recruited from the orthopaedic department in our hospital during the time from September 2012 to September 2013 (Table 1). The target number of patients was based on the quality criteria for health status questionnaires [20]. More than one hundred patients were necessary for appropriate internal consistency analysis, and more than fifty patients were required for valid reliability, validity and ceiling or floor effects analyses [20]. The study was approved by both patients and the ethics committee of our hospital. The recruited patients were required to meet the following criteria: (1) patients with operative indications were ready to receive TKA, (2) patients were able to independently complete the questionnaire without cognitive impairment (the patients with learning and memory impairment, aphasia, agnosia and dementia were assessed as cognitive impairment), (3) patients were older than 18 years, (4) the time of knee pain was longer than six weeks, and (5) laboratory tests and radiological evaluations were consistent with criteria for surgery. The exclusion criteria included the following: (1) patients had contraindications, (2) patients were unwilling to accept TKA, (3) patients had numbness of lower limbs or other neurological symptoms, (4) patients had history of operation, tumours or infection of the knee. Written informed consent was obtained from all of the subjects, the study was approved by the Local Ethics Committee of Changhai Hospital, SMMU (Shanghai, PR China) and the reference number of the ethics committee is CHEC2013-194.

**Table 1:** Characteristics of participants

Characteristics	Total (*N* = 105)
Age (yeas, mean ± SD)	63.8 ± 7.8
Sex, number (%)	
Female	59 (56 %)
Male	46 (44 %)
Side, number (%)	
Right	55 (52 %)
Left	50 (48 %)
Height (cm, mean ± SD)	166.4 ± 7.9
Weight (Kg, mean ± SD)	64.7 ± 9.1

### Instruments

NKSS is a knee-specific and self-reported questionnaire which is used to assess patients with knee diseases and it includes subjective and objective domains, the subjective domain generates an overall score ranging from 0 to 180 with a lower score representing worse knee status. To evaluate construct validity, Chinese version of OKS, the Medical Outcomes General Health Survey (SF-36) and the VAS score for pain were used to compare with the SC-NKSS. The OKS was widely used to evaluate the outcome of knee, it contains 12 items (usual level of knee pain, trouble with washing and drying, trouble with transport, walking time before severe pain, pain on standing up from sitting, limping when walking, difficulty with kneeling, pain in bed at night, work interference due to pain, sense of knee instability, doing household shopping alone, trouble with walking down stairs), with sum score ranging from 0 (worst) to 48 (best) [12]. The SF-36 which is a general health assessment questionnaire contains eight items (Physical Function (PF), Body Pain (BP), General Health (GH), Role-Physical (RP), Vitality (VT), Social Functioning (SF), Role-Emotional (RE) and Mental Health (MH). Each domain’s score ranges from 0 (worst) to 100 (best). The VAS is widely used by surgeons to measure patient’s pain level was evaluated through a 100-mm line ranging from “no pain” (at the left end) to “worst pain” (at the right end) [13, 14]. The Chinese version of NKSS, OKS, SF-36 and VAS were completed by patients at the orthopaedic inpatient or outpatient departments of our hospital.

### Score distribution and acceptability

Data acceptability was based on these criteria: normal distribution, no significant floor or ceiling effects (i.e. less than 15 % of participants achieved the highest or lowest scores). The time needed to complete the questionnaire was also recorded. No difficulties answering the questions, and no issues with missing or multiple responses.

### Reliability

The test-retest reliability of the SC-NKSS was assessed by 50 patients who were randomly selected from the sample of 105 patients. One week later, they were asked to complete the questionnaire again under similar condition and return it after they finished. The intraclass correlation coefficient (ICC) (two-way random effects model) was calculated to quantify test-retest reliability [20]. A value ICC of >0.7 was taken to indicate good reliability, and a value greater than 0.80 indicated excellent reliability [21, 22]. Internal consistency reliability was evaluated by using the Cronbach alpha coefficient, and a value greater than 0.70 was regarded as satisfactory [20, 23].

### Validity

Until now, there has been no so-called “gold standard” to optimally reflect the status of the knee [15, 16]. Simplified Chinese versions of the OKS, VAS and the SF-36 have been widely used in mainland China, and their validity and reliability have been rigorously tested [12–14, 24]. The construct validity of the SC-NKSS was assessed by calculating Pearson’s coefficient among the simplified Chinese versions of the NKSS, OKS, VAS and SF-36. The corresponding SC-NKSS subscales were expected to correlate well with the OKS, SF-36 and VAS. The correlations were judged as poor (*r* = 0–0.20), fair (0.21–0.40), moderate (0.41–0.60), very good (0.61–0.80) or excellent (0.81–1.0) [23]. The corresponding SC-NKSS subscales were expected to correlate well with the OKS, SF-36 and VAS.

## Results

### Cross-cultural adaptation

“Leisure recreational activities” are always understood as activities of playing card or watching TV and so on, in mainland China, these activities always require people to sit indoor. Therefore, the expert committee reached a consensus to use “go for a walk” instead of “leisure recreational activities” in our study. The distance measure of “a block” is unpopular in mainland China, so are “Inch” and “LBS”, therefore, the expert committees reached a consensus to use “100 m” instead of “a block”, centimetre instead of “inch”,“kilogram” instead of “LBS” in our study.

### Score distribution and acceptability

There were no floor or ceiling effects in the target population, which indicated a good distribution for the SC-NKSS. The average completing questionnaires time was 428.7 ± 24.2 s. All participants completed the questionnaire without any difficulties and no issues with missing or multiple responses.

### Reliability

Fifty participants completed the questionnaire again. Mean score of the retest was 82.08 ± 18.01, which was similar to the former result (78.42 ± 16.07). The ICC between the two sessions was 0.92 (95 % confidence interval 0.86, 0.95), which demonstrated exceptional test-retest reliability. We also found that the Cronbach alpha coefficient was 0.90 (0.71–0.85 for each subscale), which indicated excellent internal consistency for the overall SC-NKSS (Table 2).

**Table 2:** Correlation of each item and total SC-NKSS scores

	Mean ± SD	Item-total correlation	Cronbach’s alpha if item deleted
Syptom modified	6.75 ± 2.61	0.709	0.639
Satisfaction score	12.10 ± 4.42	0.820	0.504
Expectation score	10.85 ± 1.88	0.777	0.665
Functional activity score	40.95 ± 12.74	0.847	0.788

### Validity

Pearson’s coefficients are shown in table 3. The coefficient was −0.78 (95 % CI, −0.85, −0.69) between the SC-NKSS and OKS, -0.70 (95 % CI, −0.60, −0.78) between the SC-NKSS and VAS. These data demonstrate that the SC-NKSS strongly correlated with both OKS and VAS. The correlations between the SC-NKSS and the PF, BP, GH of the SF-36 (0.74, 95 % CI, 0.63, 0.81; 0.63, 95 % CI, 0.46, 0.76; 0.51, 95 % CI, 0.28, 0.67) were strong. However, the SC-NKSS exhibited weak correlations with RP (0.46, 95 % CI, 0.32, 0.59), VT (0.25, 95 % CI, 0.05, 0.43), SF (0.38, 95 % CI, 0.17, 0.54), RE (0.24, 95 % CI, 0.03, 0.44) and MH (0.27, 95 % CI, 0.05, 0.46) of the SF-36.

**Table 3:** Pearson correlations among the SC-NKSS, OKS, VAS and SF-36 (*n* = 105)

	SC-NKSS	OKS
OKS	−0.78^b^ (−0.85,−0.69)	
VAS	−0.70^b^ (−0.60,−0.78)	0.74^b^ (0.63, 0.82)
SF-36		
PF	0.74^b^ (0.64, 0.81)	−0.69^b^ (−0.79,−0.54)
RP	0.46^b^ (0.32, 0.59)	−0.42^b^ (−0.56,−0.26)
BP	0.63^b^ (0.46, 0.76)	−0.58^b^ (−0.71,−0.40)
GH	0.51^b^ (0.28, 0.67)	−0.51^b^ (−0.66,−0.33)
VT	0.25^b^ (0.05, 0.43)	−0.33^b^ (−0.49,−0.12)
SF	0.38^b^ (0.17, 0.54)	−0.46^b^ (−0.60,−0.27)
RE	0.24^a^ (0.03, 0.44)	−0.32^b^ (−0.50,−0.10)
MH	0.27^b^ (0.05, 0.46)	−0.29^b^ (−0.47,−0.08)

^a^Correlation is significant at the 0.01 level (2-tailed)

^b^Correlation is significant at the 0.05 level (2-tailed)

*OKS* Oxford knee score, *VAS* visual analogue scale, *SF-36* 36-item short form health survey, *PF* physical functioning, *RP* role-physical, *BP* bodily pain, *GH* general health, *VT* vitality, *SF* social functioning, *RE* role-emotional, *MH* mental health

## Discussion

The Knee Society Clinical Rating System appeared in 1989 and has become the most popular method for tracking and reporting outcomes of TKA [1, 8]. However, as technology and patient population have changed, defects in this scoring system have emerged. First, the number of young and active patients receiving TKA has increased rapidly. Patients expect better functional reconstruction and recreational activity, and they are unwilling to accept physical limitations [25, 26]. The Knee Society Clinical Rating System was focused on objective parameters, neglecting the patients’ subjective experiences [11, 15, 16, 27–29]. Second, many other questionnaires, including the OKS, VAS and SF-36, have frequently been used to evaluate outcomes of TKA, and they have received positive responses. The defects and ambiguities of the Knee Society Clinical Rating System challenge its utility and validity in TKA evaluation. Recently, the NKSS was developed and has been demonstrated to have good reliability and validity in the United States, Canada and The Netherlands. Our study has tested the reliability and validity of the simplified Chinese new Knee Society Scoring System (SC-NKSS). Despite differences in culture and lifestyle, the results were excellent compared with the English and Dutch versions.

There were no ceiling or floor effects in our study, which demonstrated that the distribution of the SC-NKSS was satisfactory. All patients in our study completed the questionnaire without any difficulties, which indicated the questionnaire had good cultural acceptability.

In our study, the interval time between the first and second test was 1 week. This was suitable to assess the test-retest reliability, because it was long enough to prevent recall but short enough to ensure that clinical changes had not occurred [20, 21]. The high value of the ICC (0.92) indicated excellent reproducibility, similar to the Dutch version of the NKSS [9]. The high Cronbach’s alpha coefficient (0.71–0.85) indicated a good-to-excellent internal consistency between different domains of the SC-NKSS, which is comparable with the original English version (0.68–0.95) [3] and Dutch version (0.84–0.96) [2].

The OKS was designed to evaluate the pain and physical function of patients who had undergone TKA, and the VAS objectively reflects the patients’ subjective perception of pain. Previous studies have demonstrated the validity and reliability of the simplified Chinese versions of the OKS and VAS [12–14]; therefore, the OKS and VAS scales were used as comparative criteria. In our study, we found the Pearson’s correlation coefficient was −0.78 between the SC-NKSS and OKS, −0.70 between the SC-NKSS and VAS. The strong correlation among the SC-NKSS, OKS and VAS indicated good construct validity for the SC-NKSS, similar to the English version of the NKSS [8].

The construct validity was also assessed by comparing the SC-NKSS with the SF-36 subscale. The results demonstrated that correlations between the SC-NKSS and the PF, BP and GH of SF-36 were significant. As expected, the correlations between the SC-NKSS and the RP, VT, SF, RE and MH were weak. These results are similar to the construct validity of NKSS of the Dutch version [9], demonstrating that the SC-NKSS has appropriate construct validity.

We also notice that there are some limitations in our study. First, we did not examine the responsiveness of SC-NKSS, therefore, the SC-NKSS couldn’t completely reflect improvement of patients who receivedTKA, and we plan to address it in a subsequent prospective study. Second, all patients in the sample had severe knee osteoarthritis and were awaiting TKA. Patients with mild to moderate knee osteoarthritis were not included in the study, which might result in a lower score. Third, most of our patients were from the south of China, which may not adequately represent the whole Chinese population, and we plan to make a multiple centre study in the subsequent study.

## Conclusion

The English version of the NKSS has been translated into a simplified Chinese version and has been shown to be reliable, valid and internally consistent. The SC-NKSS is easy to understand and complete. Our results suggest that the SC-NKSS is a good method for the surgeon to assess the expectations, satisfaction and physical activities of patients before and after TKA. However, its validity and reliability need further research with a larger population.

## Additional file

Additional file 1:
**Simplified Chinese version of SC-NKSS. **(PDF 584 kb)

## Abbreviations

NKSS: New Society Knee Scoring System
SC-NKSS: Simplified Chinese New Society Knee Scoring System
OKS: Oxford knee score
VAS: Visual analog scale
OA: Osteoarthritis
TKA: Total knee arthroplasty
ROM: Range of motion
SF-36: The medical outcomes general health survey
PF: Physical function
BP: Body pain
GH: General health
RP: Role-physical
VT: Vitality
SF: Social functioning
RE: Role-emotional
MH: Mental health
ICC: Intraclass correlation coefficient
