# Supplementary material for: Translation and validation of the simplified Chinese new Knee Society Scoring System
Source: BMC Musculoskelet Disord. 2015 Dec 21;16:391. doi: 10.1186/s12891-015-0854-1 (PMC4687130; doi:10.1186/s12891-015-0854-1)
Supplement: Additional file 1: — Simplified Chinese version of SC-NKSS. (PDF 584 kb) [file 12891_2015_854_MOESM1_ESM.pdf]

术前膝关节社会评分

<table>
<tr><td colspan="2" align="center">患者基本信息（患者完成）</td></tr>
<tr><td colspan="2">1-填写时间　　　　　　2-出生日期　　　　时间格式
□□/□□/□□　　　　□□/□□/□□　　　月/日/年
3-身高（厘米）　　　4-体重（千克）　　5-性别
__________ cm　　　________ Kg　　　○ 男　　○ 女</td></tr>
<tr><td colspan="2">6-有症状的膝关节（如果双膝都准备手术，则双侧膝关节各填写一份）
○ 左　　○ 右</td></tr>
<tr><td colspan="2">7-民族 _______</td></tr>
<tr><td colspan="2">8-请说明你所希望的手术时间及手术医生名字</td></tr>
<tr><td>日期 □□/□□/□□
时间格式：月/日/年</td><td>手术医生名字 ____________</td></tr>
<tr><td colspan="2">9-初次膝关节置换还是翻修
○ 初次　　　○ 翻修</td></tr>
<tr><td colspan="2" align="center">医生完成部分</td></tr>
<tr><td colspan="2">10-功能受限分类（使用以下代码）　□</td></tr>
<tr><td>A 单侧膝关节炎</td><td>C1 TKR 但远期关节炎影响移动</td></tr>
<tr><td>B1 一侧 TKA，对侧膝关节炎</td><td>C2 TKR 但身体状况影响移动</td></tr>
<tr><td>B2 双侧 TKA</td><td>C3 单侧或双侧 TKR 伴有单侧或双侧 THR</td></tr>
</table>

客观的膝关节指标（医生完成）

力线
1-解剖学力线：站立前后侧位 X 线片　　最大值 25 分<br>中立位：2-10° 外翻 （25'）<br>内翻：<2° 内翻 （-10'）<br>外翻：>10° 外翻 （-10'）<br>☐

不稳定性
2-内/外侧不稳定性：完全伸直位测量　　最大值 15 分<br>无 （15'）　轻微或<5mm （15'）<br>中度或 5mm (5')　重度或>5mm (0')<br>☐<br>3-前/后不稳定性：屈曲 90° 测量　　最大值 10 分<br>无 （10'）　中度<5mm (5')<br>中度>5mm (0')<br>☐

关节活动
4-活动范围（扣分项目，每 5° 为 1 分）☐<br>屈曲挛缩　　扣分值<br>1-5° （-2'）　6-10 （-5'）<br>11-15° （-10'）　>15° （-15'）<br>☐<br>伸直受限　　扣分值<br><10° （-5'）　10-20° （-10'）<br>>20° （-15'）<br>☐

症状（患者完成）	
1-走路时的疼痛程度<br>0 | 1 | 2 | 3 | 4 | 5 | 6 | 7 | 8 | 9 | 10<br>无疼痛　严重疼痛	（10 分）<br>☐
2-走楼梯或爬坡时疼痛程度<br>0 | 1 | 2 | 3 | 4 | 5 | 6 | 7 | 8 | 9 | 10<br>无疼痛　严重疼痛	（10 分）<br>☐
3-你感觉你的膝关节“正常”吗？<br>〇经常（5’）　〇有时（3’）　〇从来没有过（0’）	（5 分）<br>☐

最大总分值（25 分） ☐

患者满意度（患者完成）				
1-目前，坐下时，你对膝关节的疼痛水平满意吗？（8 分）				
〇非常满意（8’）	〇满意（6’）	〇比较满意（4’）	〇不满意（2’）	〇非常不满意（0’）
2-目前，躺床上时，你对膝关节的疼痛水平满意吗？（8 分）				
〇非常满意（8’）	〇满意（6’）	〇比较满意（4’）	〇不满意（2’）	〇非常不满意（0’）
3-目前，起床时，你对膝关节的功能满意吗？（8 分）				
〇非常满意（8’）	〇满意（6’）	〇比较满意（4’）	〇不满意（2’）	〇非常不满意（0’）
4-目前，做轻微家务活时，你对膝关节的功能满意吗？（8 分）				
〇非常满意（8’）	〇满意（6’）	〇比较满意（4’）	〇不满意（2’）	〇非常不满意（0’）

5-目前，散步时，你对膝关节的功能满意吗？　　　（8 分）
〇非常满意（8’） 〇满意（6’） 〇比较满意（4’） 〇不满意（2’） 〇非常不满意（0’）

最大总分值（40’）

患者的期望（患者完成）
完成膝关节置换术后你有什么样的期待？<br>1-你期待膝关节置换术后会减轻膝关节的疼痛吗？　（5′） 〇 不，一点也不　（1′）　〇 是的，一点点　（2′）<br>〇 是的，有一些　（3′）　〇 是的，比较期待　（4′）<br>〇 是的，非常期待（5′）
2-你期待膝关节置换术后能完成日常生活中的正常活动吗？（5′）<br>〇 不，一点也不　（1′）　〇 是的，一点点　（2′）<br>〇 是的，有一些　（3′）　〇 是的，比较期待　（4′）<br>〇 是的，非常期待（5′）
3-你期待膝关节置换术后能完成休息、娱乐或体育活动吗？（5′）<br>〇 不，一点也不　（1′）　〇 是的，一点点　（2′）<br>〇 是的，有一些　（3′）　〇 是的，比较期待　（4′）<br>〇 是的，非常期待（5′）

最大总分（15′）

功能活动（患者完成）

1-你可以行走不需要任何帮助吗？（如手杖、拐杖或轮椅） （0′）   ○ 是 ○ 不是
2-如果不是，需要使用以下哪些辅助物？ （-10′）   1.轮椅（-10′） 2.助步器（-8′） 3.两根拐杖（-8′）   4.两根手杖(-6′) 5 一根拐杖(-4′) 6.一根手杖(-4′) ☐   7.护膝或者膝关节支具（-2′）   其他 ____________________
3-你使用这些辅助物是因为你的膝关节吗？ （0′）   ○ 是 ○ 不是
4.由于膝关节感觉不适而坐下前，你能站多长时间（在有或没有辅助物的情况下）？ （15′）   1.不能站立（0′） 2.0-5 分钟（3′） 3.6-15 分钟（6′） ☐   4.16-30 分钟（9′） 5.31-60 分钟（12′） 6.>1h（15′）
5-由于膝关节不适而停下来之前，你能走多长时间（有或没有辅助物的情况下）？ （15′）   1.不能站立（0′） 2.0-5 分钟（3′） 3.6-15 分钟（6′） ☐   4.16-30 分钟（9′） 5.31-60 分钟（12′） 6.>1h（15′）

最大分值（30 分′） ☐

活动标准（30′）

在以下的每一个活动中你的膝关节困扰你多少？	没有困扰（5′）	轻微（4′）	中等（3′）	严重（2′）	非常严重（1′）	不能做（因为膝关节）（0′）	从来不做这个	
1-不平地面上行走	○	○	○	○	○	○	○	
2-转动或旋转腿	○	○	○	○	○	○	○	
3-向上或向下爬一段楼梯	○	○	○	○	○	○	○	
4-不需要手从矮沙发或椅子上起立	○	○	○	○	○	○	○	
5-进出小汽车	○	○	○	○	○	○	○	
6-侧移	○	○	○	○	○	○	○	

最大分值（30′）

进一步活动（25′）

1-爬梯子或踏凳	○	○	○	○	○	○	○	
2-提购物袋走100米	○	○	○	○	○	○	○	
3-下蹲	○	○	○	○	○	○	○	
4-下跪	○	○	○	○	○	○	○	
5-跑	○	○	○	○	○	○	○	

最大分值（25′）

任意活动

请挑选以下 3 种你认为对你最重要的活动<br><br>（请不要写下额外的活动）

娱乐活动	锻炼和健身活动
☐游泳	☐举重
☐高尔夫（18 杆）	☐腿扩展
☐公路自行车骑行（>30 分钟）	☐楼梯爬行
☐园艺工作	☐任意骑自行车/旋转
☐打保龄球	☐腿推举
☐球拍类运动（如网球、回力网球等）	☐慢跑
☐距离行走	☐空中漫步机
☐跳舞/芭蕾舞	☐有氧运动
☐拉伸运动（拉伸你的肌肉）	

请把以上已选的三种活动写入一下的空格中

在以下的每一个活动中你的膝关节困扰你多少？

活动（写上从上面的列表中选 3 种活动）	没有困扰（5′）	轻微（4′）	中等（3′）	严重（2′）	非常严重（1′）	不能做（因为膝关节）（0′）	从来不做这个	
1-	○	○	○	○	○	○	○	
2-	○	○	○	○	○	○	○	
3-	○	○	○	○	○	○	○	

最大分值（30′） ☐

最大总分值（100′） ☐

术后膝关节社会评分

<table>
<tr><td colspan="2">患者基本信息（患者完成）</td></tr>
<tr><td colspan="2">1-填写时间 □□/□□/□□　2-出生日期 □□/□□/□□　时间格式 月/日/年
3-身高（厘米）___________ cm　4-体重（千克）_________ Kg　5-性别 ○ 男 ○ 女</td></tr>
<tr><td colspan="2">6-有症状的膝关节（如果双膝都准备手术，则双侧膝关节各填写一份）
○ 左　○ 右</td></tr>
<tr><td colspan="2">7-民族 ________</td></tr>
<tr><td colspan="2">8-请说明你所希望的手术时间及手术医生名字</td></tr>
<tr><td>日期 □□/□□/□□
时间格式：月/日/年</td><td>手术医生名字 _____________</td></tr>
<tr><td colspan="2">9-初次膝关节置换还是翻修
○ 初次　○ 翻修</td></tr>
<tr><td colspan="2">医生完成部分</td></tr>
<tr><td colspan="2">10-功能受限分类（使用以下代码）□</td></tr>
<tr><td>A 单侧膝关节炎</td><td>C1 TKR 但远期关节炎影响移动</td></tr>
<tr><td>B1 一侧 TKA，对侧膝关节炎</td><td>C2 TKR 但身体状况影响移动</td></tr>
<tr><td>B2 双侧 TKA</td><td>C3 单侧或双侧 TKR 伴有单侧或双侧 THR</td></tr>
</table>

客观的膝关节指标（医生完成）

力线

1-解剖学力线：站立前后侧位 X 线片　　最大值 25 分

中立位：2-10° 外翻 （25'）

内翻：<2° 内翻 （-10’）

外翻：>10° 外翻 （-10’）

不稳定性

2-内/外侧不稳定性：完全伸直位测量　　最大值 15 分

无	（15’）	轻微或<5mm	（15’）
中度或 5mm	(5’)	重度或>5mm	(0’)

3-前/后不稳定性：屈曲 90° 测量　　最大值 10 分

无	（10’）	中度<5mm	(5’)
中度>5mm	(0’)		

关节活动

4-活动范围（扣分项目，每 5° 为 1 分）

屈曲挛缩　　扣分值

1-5°	（-2’）	6-10	（-5’）
11-15°	（-10’）	>15°	（-15’）

伸直受限　　扣分值

<10°	（-5’）	10-20°	（-10’）
>20°	（-15’）		

症状（患者完成）	
1-走路时的疼痛程度<br>0 1 2 3 4 5 6 7 8 9 10<br>无疼痛 严重疼痛	（10 分）<br>☐
2-走楼梯或爬坡时疼痛程度<br>0 1 2 3 4 5 6 7 8 9 10<br>无疼痛 严重疼痛	（10 分）<br>☐
3-你感觉你的膝关节“正常”吗？<br>〇经常（5’） 〇有时（3’） 〇从来没有过（0’）	（5 分）<br>☐

最大总分值（25分） ☐

患者满意度（患者完成）				
1-目前，坐下时，你对膝关节的疼痛水平满意吗？				（8 分）
〇非常满意（8’）	〇满意（6’）	〇比较满意（4’）	〇不满意（2’）	〇非常不满意（0’）
2-目前，躺床上时，你对膝关节的疼痛水平满意吗？				（8 分）
〇非常满意（8’）	〇满意（6’）	〇比较满意（4’）	〇不满意（2’）	〇非常不满意（0’）
3-目前，起床时，你对膝关节的功能满意吗？				（8 分）
〇非常满意（8’）	〇满意（6’）	〇比较满意（4’）	〇不满意（2’）	〇非常不满意（0’）
4-目前，做轻微家务活时，你对膝关节的功能满意吗？				（8 分）
〇非常满意（8’）	〇满意（6’）	〇比较满意（4’）	〇不满意（2’）	〇非常不满意（0’）

5-目前，散步时，你对膝关节的功能满意吗？（8 分）<br>〇非常满意（8’） 〇满意（6’） 〇比较满意（4’） 〇不满意（2’） 〇非常不满意（0’）

最大总分值（40’）

患者的期待（患者完成）
与你行膝关节置换前的期望值做比较<br>1-我对缓解疼痛的期待值（5′）<br>〇太高-比期待值低很多（1′） 〇太高-比期待值低一些（2′）<br>〇正好-满足期待值（3′） 〇太低-比期待值高一些（4′）<br>〇太低-比期待值高很多（5′）
2-我对能完成日常生活中的正常活动的期待值（5′）<br>〇太高-比期待值低很多（1′） 〇太高-比期待值低一些（2′）<br>〇正好-满足期待值（3′） 〇太低-比期待值高一些（4′）<br>〇太低-比期待值高很多（5′）
3-我对完成休息、娱乐或体育活动的期待值（5′）<br>〇太高-比期待值低很多（1′） 〇太高-比期待值低一些（2′）<br>〇正好-满足期待值（3′） 〇太低-比期待值高一些（4′）<br>〇太低-比期待值高很多（5′）

最大总分（15′）

功能活动（患者完成）

1-你可以行走不需要任何帮助吗？（如手杖、拐杖或轮椅）<br>○ 是　　　　○ 不是	(0′)
2-如果不是，需要使用以下哪些辅助物？<br>1.轮椅（-10′）　2.助步器（-8′）　3.两根拐杖（-8′）<br>4.两根手杖(-6′)　5 一根拐杖(-4′)　6-一根手杖(-4′)<br>7.护膝或者膝关节支具（-2′）<br>其他 ______________________________	(-10′)<br>☐
3-你使用这些辅助物是因为你的膝关节吗？<br>○ 是　　　　○ 不是	(0′)
4.由于膝关节感觉不适而坐下前，你能站多长时间（在有或没有辅助物的情况下）？<br>1.不能站立（0′）　2.0-5 分钟（3′）　3.6-15 分钟（6′）<br>4.16-30 分钟（9′）　5.31-60 分钟（12′）　6.>1h（15′）	(15′)<br>☐
5-由于膝关节不是而停下来之前，你能走多长时间（有或没有辅助物的情况下）？<br>1.不能站立（0′）　2.0-5 分钟（3′）　3.6-15 分钟（6′）<br>4.16-30 分钟（9′）　5.31-60 分钟（12′）　6.>1h（15′）	(15′)<br>☐

最大分值（30 分′）☐

活动标准（30′）

在以下的每一个活动中你的膝关节困扰你多少？	没有困扰（5′）	轻微（4′）	中等（3′）	严重（2′）	非常严重（1′）	不能做（因为膝关节）（0′）	从来不做这个	
1-不平地面上行走	○	○	○	○	○	○	○	
2-转动或旋转腿	○	○	○	○	○	○	○	
3-向上或向下爬一段楼梯	○	○	○	○	○	○	○	
4-不需要手从矮沙发或椅子上起立	○	○	○	○	○	○	○	
5-进出小汽车	○	○	○	○	○	○	○	
6-侧移	○	○	○	○	○	○	○	

最大分值（30′）

进一步活动（25′）

1-爬梯子或踏凳	○	○	○	○	○	○	○	
2-提购物袋走100米	○	○	○	○	○	○	○	
3-下蹲	○	○	○	○	○	○	○	
4-下跪	○	○	○	○	○	○	○	
5-跑	○	○	○	○	○	○	○	

最大分值（25′）

任意活动

请挑选以下 3 种你认为对你最重要的活动<br><br>（请不要写下额外的活动）

娱乐活动	锻炼和健身活动
☐游泳	☐举重
☐高尔夫（18 杆）	☐腿扩展
☐公路自行车骑行（>30 分钟）	☐楼梯爬行
☐园艺工作	☐任意骑自行车/旋转
☐打保龄球	☐腿推举
☐球拍类运动（如网球、回力网球等）	☐慢跑
☐距离行走	☐空中漫步机
☐跳舞/芭蕾舞	☐有氧运动
☐拉伸运动（拉伸你的肌肉）	

请把以上已选的三种活动写入一下的空格中

在以下的每一个活动中你的膝关节困扰你多少？

活动（写上从上面的列表中选 3 种活动）	没有困扰（5′）	轻微（4′）	中等（3′）	严重（2′）	非常严重（1′）	不能做（因为膝关节）（0′）	从来不做这个	
1-	○	○	○	○	○	○	○	
2-	○	○	○	○	○	○	○	
3-	○	○	○	○	○	○	○	

最大分值（30′）☐

最大总分值（100′）☐
